# Identifying and Validating a Combined mRNA and MicroRNA Signature in Response to Imatinib Treatment in a Chronic Myeloid Leukemia Cell Line

**DOI:** 10.1371/journal.pone.0115003

**Published:** 2014-12-15

**Authors:** Steven Bhutra, Divya Lenkala, Bonnie LaCroix, Meng Ye, R. Stephanie Huang

**Affiliations:** 1 Pritzker School of Medicine, University of Chicago, Chicago, Illinois, United States of America; 2 Department of Medicine, University of Chicago, Chicago, Illinois, United States of America; 3 The Affiliated Hospital, School of Medicine, Ningbo University, Ningbo, Zhejiang, China; University of North Carolina at Chapel Hill, United States of America

## Abstract

Imatinib, a targeted tyrosine kinase inhibitor, is the gold standard for managing chronic myeloid leukemia (CML). Despite its wide application, imatinib resistance occurs in 20–30% of individuals with CML. Multiple potential biomarkers have been identified to predict imatinib response; however, the majority of them remain externally uncorroborated. In this study, we set out to systematically identify gene/microRNA (miRNA) whose expression changes are related to imatinib response. Through a Gene Expression Omnibus search, we identified two genome-wide expression datasets that contain expression changes in response to imatinib treatment in a CML cell line (K562): one for mRNA and the other for miRNA. Significantly differentially expressed transcripts/miRNAs post imatinib treatment were identified from both datasets. Three additional filtering criteria were applied 1) miRbase/miRanda predictive algorithm; 2) opposite direction of imatinib effect for genes and miRNAs; and 3) literature support. These criteria narrowed our candidate gene-miRNA to a single pair: *IL8* and miR-493-5p. Using PCR we confirmed the significant up-regulation and down-regulation of miR-493-5p and *IL8* by imatinib treatment, respectively in K562 cells. In addition, *IL8* expression was significantly down-regulated in K562 cells 24 hours after miR-493-5p mimic transfection (p = 0.002). Furthermore, we demonstrated significant cellular growth inhibition after *IL8* inhibition through either gene silencing or by over-expression of miR-493-5p (p = 0.0005 and p = 0.001 respectively). The *IL8* inhibition also further sensitized K562 cells to imatinib cytotoxicity (p<0.0001). Our study combined expression changes in transcriptome and miRNA after imatinib exposure to identify a potential gene-miRNA pair that is a critical target in imatinib response. Experimental validation supports the relationships between *IL8* and miR-493-5p and between this gene-miRNA pair and imatinib sensitivity in a CML cell line. Our data suggests integrative analysis of multiple omic level data may provide new insight into biomarker discovery as well as mechanisms of imatinib resistance.

## Introduction

Chronic myelogenous leukemia (CML) is a clonal myeloproliferative neoplasm of mature and maturing granulocytes resulting from the translocation of chromosomes 9 and 22 t(9;22)(q34:q11) [1]. This fusion generates the shortened 22q known as the Philadelphia (Ph) chromosome and the new fusion oncogene *BCR-ABL1,* a constitutively active tyrosine kinase that can be selectively targeted by imatinib and other tyrosine kinase inhibitors (TKIs) [2], [3]. Imatinib is a small molecule inhibitor that semi-competitively binds the ATP-binding site of BCR-ABL, thus preventing the conformational switch to its active form and inhibiting enzymatic activity, interfering with downstream signal transduction [4]. Treatment with imatinib alone in the early-chronic phase of CML shows an 88% overall survival rate after five years compared to 57% with previous nonspecific treatments including interferon and hydroxyurea, with far fewer side effects [5], [6]. Although, imatinib prolongs survival, it does not cure the disease. The success of the drug as a front-line therapy in CML has been tempered by latent problems such as disease persistence or relapse in nearly 20–30% of all CML patients [7]. Moreover, imatinib has been cited as one of the first exceptionally expensive cancer drugs, costing nearly $100,000 per year per patient [8]. Given the huge financial burden and sporadically temporary management, it is crucial to identify molecular mechanisms of imatinib resistance in order to individually tailor imatinib therapy and spare those non-responders from unnecessary side effects and economic burden.

There have been dozens of studies which predict imatinib response based on molecular components in both *in vitro* and *in vivo* models [9]–[27]; however, the majority of these studies remain externally uncorroborated [9]–[12], [26]. This lack of reproducibility is in part due to the lack of comprehensive evaluation of biomarkers on a global scale [28]. Over the past decade, increasing accessibility of high-throughput sequencing and mass spectroscopy has made it possible to profile a multitude of molecular components in a cost-effective and timely way [29], [30]. The National Center for Biotechnology Information (NCBI) Gene Expression Omnibus (GEO) provides a platform to increase data sharing in the research community and to allow subsequent data mining [31]. With the advent of expansive molecular component data, the ability to integrate these data may provide a better understanding of the underlying biology of human complex traits [32].

Here, we propose a pilot integrative approach that combines gene and microRNA (miRNA) expression changes in response to imatinib in a CML cell line K562 to identify potential markers and pathways that are important in imatinib response. The rationale for focusing on K562 is based on the rich publicly available transcriptome and miRNA expression data post imatinib treatment, which provides us a tool to conduct this proof-of-concept study. Gene and miRNA expression change were used over baseline expression in order to improve the molecular level resolution. Although imatinib was designed to specifically target BCR-ABL, little is understood about the drug’s downstream effects. By focusing on expression change we hoped to uncover gene/miRNA networks that are specifically responsive to imatinib treatment. Furthermore, functional experiments were conducted to validate the role of the putative mRNA and miRNA markers in response to imatinib treatment.

## Materials and Methods

### Identify imatinib responsive gene/miRNA

**Figure 1:**
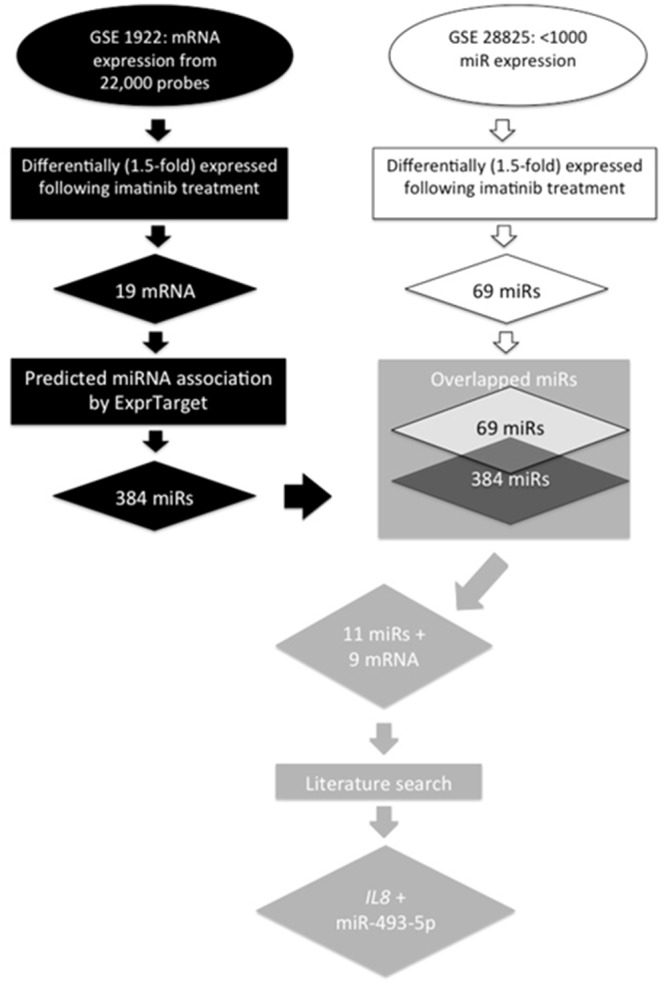
Work flow for identifying imatinib response biomarkers through an informatics approach in K562 cells.

Whole genome gene and miRNA expression levels in the K562 cell line with and without imatinib treatment were obtained from GEO. Dataset GSE1922 contains whole genome mRNA expression data after 24 hours of 1 µM imatinib treatment [33] and dataset GSE28825 contains whole genome miRNA expression after 48 hours of 0.15 µM imatinib treatment [13]. Both datasets contain appropriate no treatment controls. Detailed workflow can be found in Fig. 1. From dataset GSE1922, 19 mRNAs were selected which showed a greater than 1.5-fold change in expression level after imatinib treatment as compared to control (p value threshold <2.2×10^–6^). Similarly, 69 miRNAs were selected from dataset GSE28825 by filtering for human miRNA probes with expression levels greater than background (307 miRNA probes with expression level >0.1), removing any probes with higher than average standard deviation (239 miRNA probes), and selecting only those miRNAs which showed a greater than 1.5-fold change in expression level after imatinib treatment as compared to control. The 19 differentially expressed genes were examined for their predicted miRNA binding partners using the miRanda algorithm built on miRBase annotation (http://www.scandb.org/apps/microrna/search.html). In total, the 19 unique imatinib affected genes have 384 potential miRNA partners. These potential partners were then compared to the 69 differentially expressed miRNA from dataset GSE28825 to identify overlapping miRNA. Additional filtering criteria were applied to select only those differentially expressed mRNA and miRNA, which are negatively correlated, leaving 9 unique mRNA and 11 unique miRNA. In addition, we used literature support to further narrow down our list of gene-miRNA pairs for functional validation. Criteria used include gene/miRNA’s biological role in cancer and potential interaction with imatinib.

### Cells and materials

CML cell line K562 was obtained from ATCC (cat # CCL-243). Cryopreserved cells were thawed and grown at 37°C and 5% CO_2_ in RPMI medium supplemented with 15% fetal bovine serum, and 2% L-glutamine. Cells were passaged 3 times per week to maintain exponential growth. Imatinib mesylate (Enzo Life Sciences cat # ALX-270-492) was dissolved in DMSO to 100 mM master stock, aliquoted, and stored at −20°C.

### Effect of imatinib on selected gene and miRNA expression

K562 cells were seeded in 6-well plates at 2×10^6^ cells per well. After 24 hours, the cells were treated with 1 µM imatinib. Viable cells were counted, collected and snap frozen at 0, 24 and 48 hours after drug treatment. Cells were homogenized using QiaShredders and total RNA was isolated using Qiagen miRNeasy kit (cat # 217004). Complementary cDNA was generated using Applied Biosystems High Capactity cDNA Reverse Transcription Kit (cat # 4368813) or Exiqon Universal cDNA Synthesis Kit II (cat # 203301) for mRNA and miRNA respectively. The relative expression of *IL8* and miR-493-5p was evaluated by real time PCR (RT-PCR). Applied Biosystems Taqman Fast Advanced Master Mix (cat # 4444557) and hs00174103_m1 primer set were used for *IL8*; while Exiqon ExiLENT SYBR Green Master Mix (cat # 203402) and Exiqon primer set (cat # 205693) were used for miR-493-5p. Gene and miRNA expression levels were normalized to *B2M* and RNU6 respectively. Relative expression was quantified using the standard curve method.

### miR-493-5p effect on *IL8*

K562 cells were seeded at 5×10^5^ cells per well in a 6-well plate and transfected with 15 nM miR-493-5p mimic (Qiagen MSY0002813), AllStar Negative Control siRNA (Qiagen cat **#** SI03650318) or water using DharmaFECT Transfection Reagent 1 (Thermo Fisher Scientific cat # T-2001) according to the manufacturer’s instructions. After six hours, transfection media was removed and replaced with complete culture media. Cells were pelleted after 0, 6, 24 and 48 hours for RNA isolation, cDNA conversion and RT-PCR for miR-493-5p and *IL8*. Mean and standard deviation were calculated for the normalized quantity of each target transcript at each time point and treatment condition. Paired, two-tailed Student’s t-tests were performed comparing treatment and control conditions at each time point with p<0.05 considered statistically significant.

### Evaluate biological function of miR-493-5p over-expression or *IL8* inhibition

K562 cells were transfected with 15 nM miR-493-5p mimic, *IL8* siRNA (Qiagen cat # SI00012236), AllStar Negative Control siRNA (Qiagen cat **#** SI03650318) or water independently using DharmaFECT Transfection Reagent 1 described above. Immediately after transfection, cells were plated to assess either cellular proliferation rate or imatinib sensitivity. To measure cellular proliferation rate, cells were plated at 5,000 cells per well in a 96-well plate and CellTiter-Glo Luminescent Cell Viability Assay (Promega cat # G7570) was performed after 0, 24, 48, and 72 hours. For imatinib sensitivity measurement, transfected cells were plated in 96-well plates at 10,000 cells per well. 24 hours later, cells were treated with increasing concentrations of imatinib (0, 0.3125, 0.625, 1, 1.25, 2, and 2.5 µM). 48 hours after the addition of imatinib, 100 µL of cell mixture from each well were combined with 100 µL of CellTiter-Glo reagent. For both assays luminescence was measured using a BioTEK Synergy HT plate reader. Cellular sensitivity to imatinib was presented as percent survival in comparison to control at each concentration. Two-way ANOVA was performed to evaluate the difference among different gene/miRNA modification conditions with p<0.05 considered statistically significant.

### Explore the role of *IL8* in CML patients

To explore the role of IL8 in CML patients, we queried another GEO dataset (GSE4170)[34]. In this study, gene expression profiling was conducted in bone marrow from 91 cases of CML in chronic, accelerated, and blast phases. Furthermore, additional cases of patients who relapsed after initially successful imatinib treatment were also profiled. We compared the *IL8* expression level from the chronic CML group to those patients who were treated with imatinib at the chronic phase and later relapsed. Student t test was performed with alpha value less than 0.05 was considered statistically significant.

## Results

### Identify imatinib responsive gene/miRNA

The workflow and stepwise findings is shown in Fig. 1. Two datasets were selected from the 21 unique studies garnered through GEO search results based on treatment conditions and molecular components investigated. They are GSE1922 and GSE28825. 19 distinct mRNA and 69 distinct miRNA were found to be differentially expressed in datasets GSE1922 and GSE28825 respectively. Integrating these two datasets using the methods described above identified 12 mRNA/miRNA relationships made up of 9 unique mRNA and 11 unique miRNA (Fig. 1 and Table 1). One mRNA/miRNA pair: *IL8* and miR-493-5p, was chosen for subsequent functional validation based on literature support. For example, *IL8* expression has been shown to be under expressed in patients post imatinib treatment and miR-493-5p has been implicated in colon metastasis [35], [36], bladder cancer [37], ovarian cancer [38] and triple negative breast cancer [39]; however, the regulatory connection between this gene/miRNA pair or their functional connection to imatinib sensitivity is unknown.

**Table 1:** 12 predicted imatinib response gene-miRNA pairs based on GEO data.

Gene name	mRNA expression fold change	miR name	miRNA expression fold change
*ALAS2*	1.73	miR-500	-2.23
*ALAS2*	1.73	miR-886-3p	-1.74
*CD69*	−1.68	miR-323-3p	1.52
*COL18A1*	1.69	miR-933	−1.67
*EGR1*	−1.54	miR-493-5p	1.52
***IL8***	−**1.51**	**miR-493-5p**	**1.52**
*PDE4DIP*	−1.54	miR-877*	1.54
*PDE4DIP*	−1.54	miR-483*	1.71
*PHLDA2*	−1.6	miR-134	1.71
*RHD*	1.54	miR-19b	−1.6
*SLC30A10*	1.52	miR-335*	−4.01
*SLC30A10*	1.52	miR-193b	−1.91

Functional experiments were conducted on the bolded gene/miR pair.

### Assess relationships among imatinib treatment, *IL8* and miR-493-5p expression

**Figure 2:**
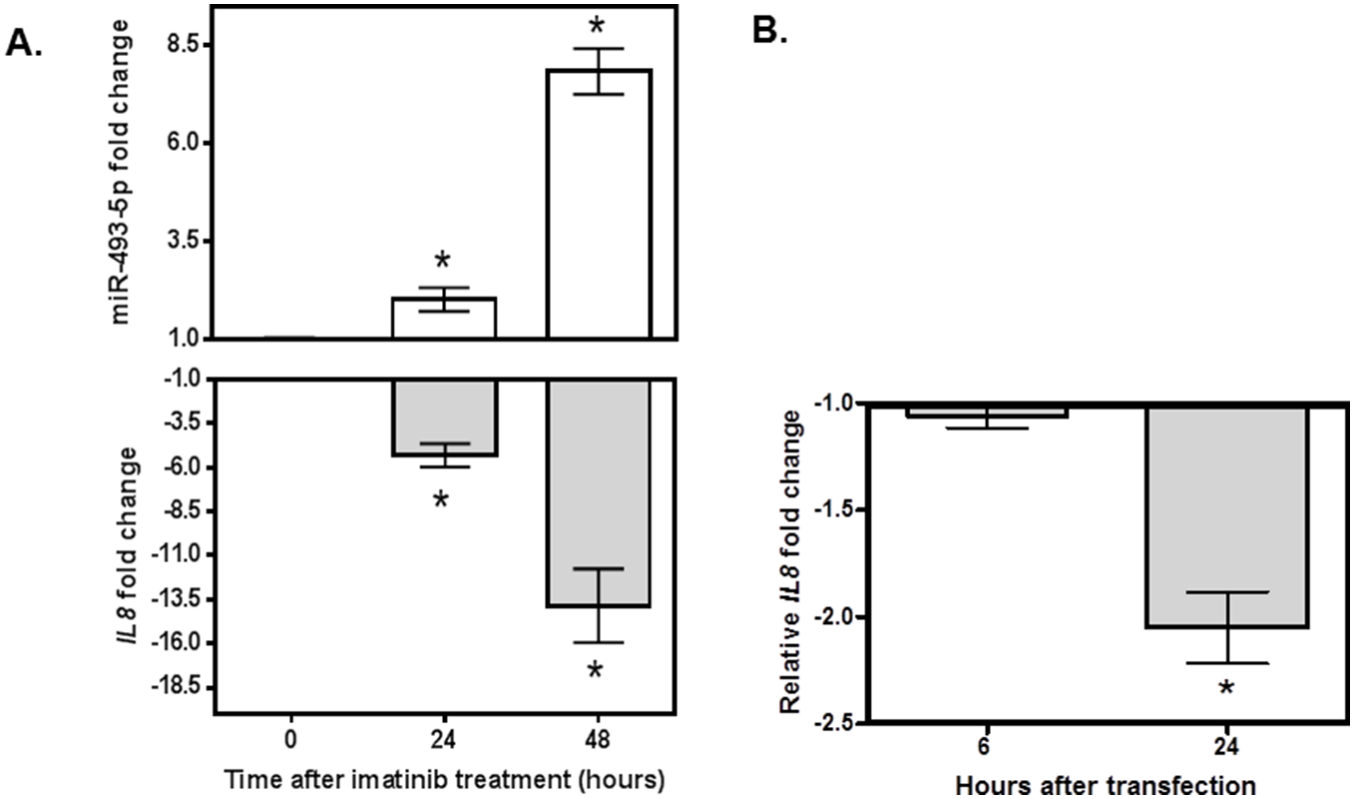
Relationships among imatinib treatment, and the expression of miR-493-5p and *IL8* in K562 cells. (**A**) Imatinib treatment (1µM for 24 and 48 hours) significantly up-regulated miR-493-5p expression and significantly down-regulated *IL8* expression in K562 cells (*p<0.05, N = 6). (**B**) *IL8* is significantly down-regulated by miR-493-5p mimic at 24 hours after transfection (*p = 0.002, N = 6). Relative fold change was calculated by first normalizing *IL8 to B2M* and miR-493-5p to *RNU6* and then comparing 6 and 24 hours data to 0 hour data.

We treated K562 cells with imatinib and measured both *IL8* and miR-493-5p expression after 24 and 48 hours. As expected, *IL8* expression was significantly down-regulated; while miR-493-5p expression was significantly up-regulated post imatinib treatment both at 24 and 48 hours (respectively p = 0.02 and p = 0.0001 for miR-493-5p, p = 0.001 and p = 0.001 for *IL8*) (Fig. 2A). In addition, after transfection with miR-493-5p mimic, significant down-regulation of *IL8* was observed at 24 hours (p = 0.002) when compared to scramble treated control (Fig. 2B). However, the inhibitory effect of miR-493-5p mimic on *IL8* expression was not observed at 48 hours post mimic transfection (p>0.05, data not shown).

### Evaluate biological function of expression modulation of miR-493-5p or *IL8*

**Figure 3:**
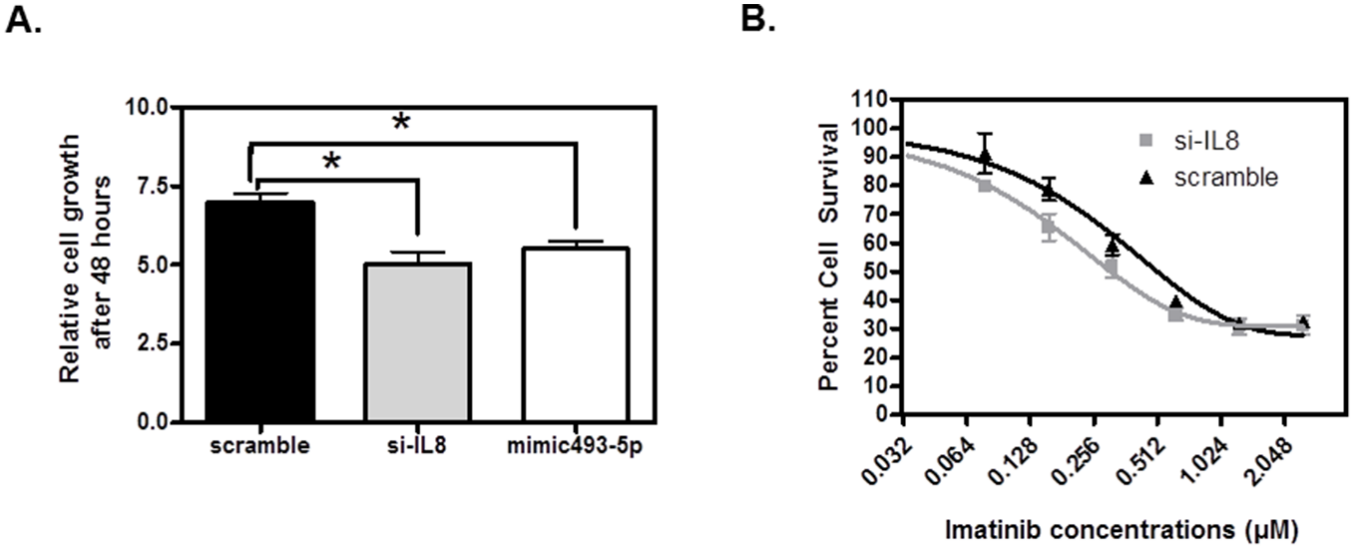
Biological consequences of *IL8* inhibition. (**A**) *IL8* silencing through siRNA and miR-493-5p mimic resulted in significantly slower cellular proliferation as measured by CellTiter-Glo at 48 hours. (*p<0.05, N = 6). Relative cell growth was calculated by normalizing fluorescence indexes of each time point to the 0 hour control. (**B**) Cellular response to imatinib treatment curves. Cellular response to imatinib was measured using CellTiter-Glo. p<0.0001 by two way ANOVA, N = 6. Percent survival was calculated by normalizing fluorescence indexes of imatinib treated cells to cells treated with vehicle and plotting on a log(2) scale.

In order to investigate the consequences of *IL8* inhibition, we transfected K562 cells with siRNA for *IL8* and miR-493-5p mimic and measured cell viability. Both *IL8* inhibition and miR-493-5p over-expression resulted in significant decrease in *IL8* mRNA expression (p<0.05 at 6, 24, and 48 hours post si-IL8; and at 6, and 24 hours post miR-493-5p mimic). Furthermore, inhibition of *IL8* resulted in marked decrease in K562 cell proliferation measured by CellTiter-Glo at 48 hours (Fig. 3A, p = 0.0005 and p = 0.001 respectively). Moreover, cytotoxicity experiments showed that K562 cells transfected with *IL8* siRNA were more sensitive to imatinib treatment (Fig. 3B, two-way ANOVA p<0.0001). Imatinib IC_50_ is 0.30 and 0.50 µM for *IL8* siRNA and scrambled control transfected K562 cells respectively. The effect of miR-493-5p overexpression did not result in a change in imatinib sensitivity in comparison to scrambled control.

### Explore the role of *IL8* in CML patients

We obtained another GEO dataset (GSE4170) [34] and compared the *IL8* expression level from 23 chronic phase CML patients to 9 of those patients who were treated with imatinib at the chronic phase and later relapsed. A significant difference in *IL8* expression was observed between these 2 groups (p = 1×10^−4^).

## Discussion

Imatinib and other TKI implementation has resulted in a major breakthrough for the treatment of CML, but resistance remains a significant clinical problem. Although it is clear that *BCR-ABL* fusion gene is a key target of imatinib, little else is known about the downstream effects of BCR-ABL or other molecular targets of imatinib in CML. Indeed, recent recognition of imatinib’s other on and off target effects have led to the testing of imatinib treatment in many other disease settings including Gastointestinal stromal tumor [40], AIDS associated Kaposi’s sarcoma [41], and systemic sclerosis [42]. Molecular response signatures based on expression change can provide both a snapshot of the downstream targets specific to a drug and clinical insight into drug efficacy and resistance mechanisms that can be used to guide patient care. Taking advantage of existing large omic datasets from GEO, we set out to develop an approach that integrates both transcriptome and epigenome changes to decipher the underlying biology of imatinib response.

By thoroughly examining two independent datasets along with incorporation of an mRNA-miRNA relationship prediction tool, we identified a set of 12 pairs of gene/miRNA relationships (consisting of 9 genes and 11 miRNAs), all of which are affected by imatinib treatment. Among them, *SLC30A10* is a known zinc binding protein imperative in autophagy [43] and has been implicated in colorectal cancer [44]. *PHLD2* is one of several genes in the imprinted gene domain of 11p15.5, which is considered to be an important tumor suppressor gene region. Alterations in this region have been associated with Wilms tumor, osterosarcoma, rhabdomyosarcoma, lung, ovarian, and breast cancer [45]. *PDE4DIP* is part of the PDGFR family and is known to mark eosinophilia and myeloproliferative disorders [46]. All these gene/miRNA pairs warrant further evaluation in relation to imatinib sensitivity in CML.

Our study followed up on one gene/miRNA pair (*IL8*/miR-493-5p) both of which are affected by imatinib treatment. *IL8* is a known proinflammatory cytokine important in the NFKB survival pathway [47]. In a previous clinical study imatinib treatment response was shown to down-regulate *IL8* expression in patients with CML [48]. Our study confirmed similar effect of imatinib in K562 cells with treatment effects lasting at least 48 hours. miR-493-5p has been reported to be differentially expressed in multiple cancers [34]. In particular, its expression is known to decrease cell growth and migration in bladder cancer by inhibiting targets *RhoC* and *FDZ4* [37]. In our study, we identified miR-493-5p as a potential mediator for *IL8* down-regulation as evidenced by 1) the miRbase miRanda algorithm prediction [49], [50]; and 2) observed significant *IL8* down-regulation after addition of miR-493-5p mimic. We speculated that imatinib treatment may up-regulate miR-493-5p expression which subsequently inhibits *IL8* expression and led imatinib sensitivity, although these sequential events remain to be tested.

By integrating two distinct high throughput expression datasets we were able to hone in on a gene/miRNA pair - *IL8*/miR-493-5p – critical for imatinib sensitivity. Our work suggests that differential inhibition of *IL8* among individuals may be a good marker for imatinib sensitivity. Furthermore, we speculate a directional relationship where imatinib can induce miR-493-5p expression which then subsequently inhibits *IL8* expression and leads to biological consequences such as cellular growth inhibition. We also demonstrate that inhibition of *IL8* in addition to imatinib may result in better disease control, which supports the need for further evaluation of IL8 inhibition as a tool to overcome imatinib resistance.
